# A Newly Synthesized Flavone from Luteolin Escapes from COMT-Catalyzed Methylation and Inhibits Lipopolysaccharide-Induced Inflammation in RAW264.7 Macrophages via JNK, p38 and NF-κB Signaling Pathways

**DOI:** 10.4014/jmb.2104.04027

**Published:** 2021-05-27

**Authors:** Lin Ye, Yang Xin, Zhi-yuan Wu, Hai-jian Sun, De-jian Huang, Zhi-qin Sun

**Affiliations:** 1 School of Pharmacy, Changzhou University, Changzhou 213164, P.R. China; 2 Department of Pharmacology, Yong Loo Lin School of Medicine, National University of Singapore, Singapore 117597, Singapore; 3 Food Science and Technology Program, Department of Chemistry, Faculty of Science, National University of Singapore, Singapore 117597, Singapore; 4 National University of Singapore (Suzhou) Research Institute, Suzhou, Jiangsu 215123, P.R. China; 5 Changzhou Second People's Hospital, Changzhou 213000, P.R. China

**Keywords:** Luteolin, 2, 2'-azobis(2-amidinopropane) dihydrochloride (AAPH), catechol-O-methyltransferases (COMT), inflammation

## Abstract

Luteolin is a common dietary flavone possessing potent anti-inflammatory activities. However, when administrated in vivo, luteolin becomes methylated by catechol-O-methyltransferases (COMT) owing to the catechol ring in the chemical structure, which largely diminishes its anti-inflammatory effect. In this study, we made a modification on luteolin, named LUA, which was generated by the chemical reaction between luteolin and 2,2'-azobis(2-amidinopropane) dihydrochloride (AAPH). Without a catechol ring in the chemical structure, this new flavone could escape from the COMT-catalyzed methylation, thus affording the potential to exert its functions in the original form when administrated in the organism. Moreover, an LPS-stimulated RAW cell model was applied to detect the anti-inflammatory properties. LUA showed much more superior inhibitory effect on LPS-induced production of NO than diosmetin (a major methylated form of luteolin) and significantly suppressed upregulation of iNOS and COX-2 in macrophages. LUA treatment dramatically reduced LPS-stimulated reactive oxygen species (ROS) and mRNA levels of pro-inflammatory mediators such as IL-1β, IL-6, IL-8 and IFN-β. Furthermore, LUA significantly reduced the phosphorylation of JNK and p38 without affecting that of ERK. LUA also inhibited the activation of NF-κB through suppression of p65 phosphorylation and nuclear translocation.

## Introduction

Inflammatory response, as a common host defense reaction against pathogen invasion or other injuries, plays a crucial role in the protection of vascularized living organisms. Normally, inflammation is accompanied by various kinds of processes, among which the innate immune system is initially activated. Macrophages are the primary innate immune cells substantially responsible for phagocytosis and antigen presentation [1, 2]. When the macrophages are stimulated by harmful factors, they can produce and release many inflammation mediators and cytokines like nitric oxide (NO), prostaglandins (PGs), tumor necrosis factor-α (TNF-α), interleukin-1β (IL-1β), and interleukin-6 (IL-6). Although moderate inflammation contributes to the elimination of microbes from tissues [3], excessive activation of inflammation has been identified to induce cell damages and even some chronic diseases based on inflammation including atherosclerosis, diabetes, rheumatoid arthritis, and cancer [4-7]. Therefore, therapeutic drugs with anti-inflammatory properties are required to alleviate damage induced by excessive inflammatory response.

**Fig. 1:**
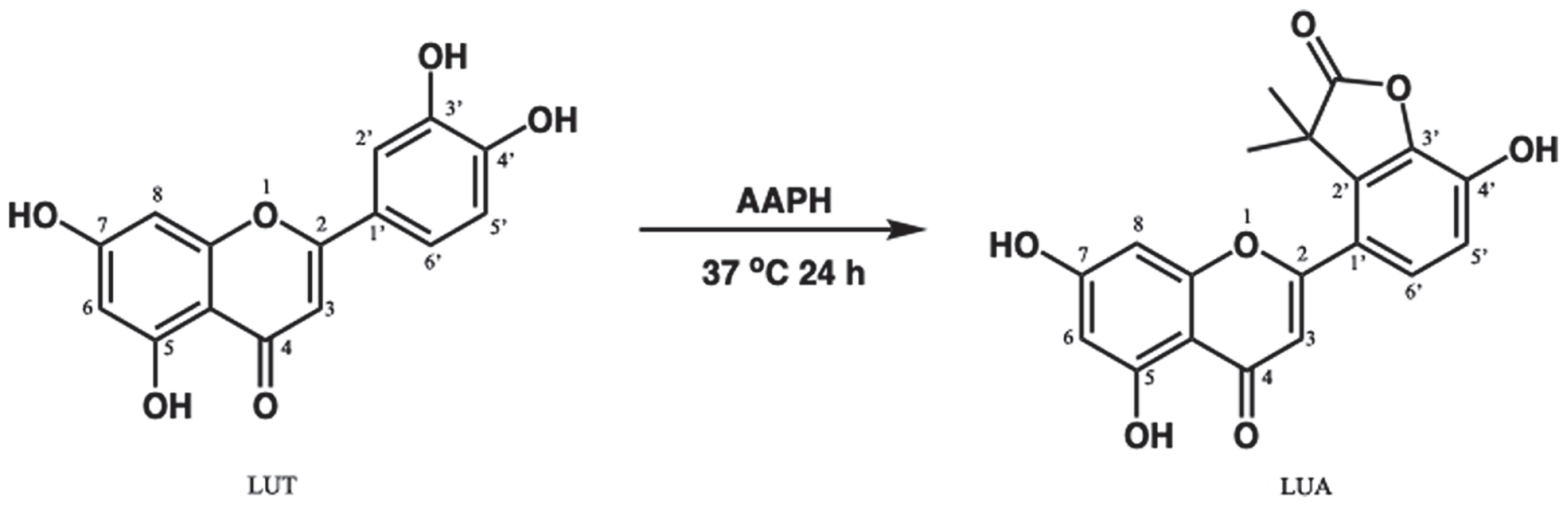
Synthetic pathway and chemical structure of LUA. LUA: LUAAPH-1.

Flavones are a class of flavonoids typically found in a variety of fruits and vegetables [8] and have been employed as anti-oxidative, anti-inflammatory, anti-microbial, anti-diabetic and immunoregulatory remedies for centuries [9-12]. Luteolin (3',4',5,7-hydroxyl-flavone, Fig. 1), a natural flavone abundant in vegetables such as cabbages, celery, carrots, broccoli, peppers, onion leaves, apple skins, and chrysanthemum flowers [13], has received extensive attention not only because of its versatile health benefits [14-17], but also for the fact that it exerts pharmacological activity at micromolar concentrations [18]. Increasing evidence has demonstrated that luteolin exhibits one of the strongest anti-inflammatory properties among flavonoids via different mechanisms both in vivo and in vitro [14, 19-21].

However, despite the intense anti-inflammation activity, the bioactivity of luteolin will decrease when administrated in vivo [22]. This may be explained partly because of the wide methylation of luteolin in the body, which is mediated by catechol-O-methyltransferases (COMT). In mammals, COMT is an enzyme ubiquitously distributed throughout the organs of the body. The highest concentration of COMT protein and activity levels is observed in the liver, followed by the kidneys and gastrointestinal tract [23-26]. COMT can catalyze the O-methylation of any substrate containing the structure of catechol, such as epinephrine, norepinephrine, and dopamine [27]. Luteolin, with a catechol ring structure, has also been identified as a substrate of COMT [28, 29]. Numerous studies have detected the methylated metabolites of luteolin, which are composed of diosmetin, chrysoeriol and their respective glucuronides conjugates [30, 31]. The methylated metabolites not only lead to the faster elimination of luteolin [22], but also have much weaker anti-inflammatory effects than luteolin [31]. However, in the presence of COMT inhibitor, methylated derivatives of luteolin are remarkably reduced, leaving more luteolin conjugates metabolites exhibiting superior anti-inflammatory activities [31]. These results demonstrate that the methylation by COMT plays a critical role in inhibiting the anti-inflammatory effects of luteolin in the body. Therefore, it is necessary to prevent luteolin from being methylated by COMT to preserve its anti-inflammatory activities. Although COMT inhibitors have been discovered, some of them are associated with lots of problems, such as cytotoxicity, low bioavailability, short-acting inhibitory profile and intestinal side effects [32-34]. For this reason, there is a requirement for safer and more efficacious approaches to improve the methylated disposition of luteolin.

To achieve this objective, we sought to optimize the structure of luteolin by chemical methods, generating a new flavone compound named LUAAPH-1(LUA), which would escape methylation by COMT and exert strong anti-inflammation effects. In vitro studies were then performed on RAW264.7 cell lines to detect the anti-inflammatory activity of the new compound.

## Materials and Methods

### Reagents and Antibodies

Lipopolysaccharide (LPS, *Escherichia coli* 0111:B4) and 2,7-dichlorofluorescein diacetate (DCFH-DA) were obtained from Sigma-Aldrich (USA). Water-soluble tetrazolium-1(WST-1) for cell proliferation analysis was from BioVision (USA). Griess reagent was supplied by Promega (USA). The antibody for COX-2, t-P65, β-actin, and GAPDH was from Santa Cruz Biotechnology (USA). The IL-1β antibody was from Abcam (USA). All the other antibodies were obtained from Cell Signaling Technology (USA). Any other reagent was obtained from Sigma-Aldrich unless specifically stated. Luteolin was supplied by Nanjing Plant Origin Biological Technology Co., Ltd. Lastly, 2,2’-azobis(2-amidinopropane) dihydrochloride (AAPH) was obtained from Merck & Co., Inc.(USA).

### The Synthesis Procedure of the New Flavone

LUA was synthesized and identified in our laboratory previously. Briefly, 2,2’-azobis(2-amidinopropane) dihydrochloride (AAPH, 244 mg, 0.9 mmol) and luteolin (42.9 mg, 0.15 mmol) were dissolved in 75 mM phosphate buffer (pH 7.4) in a 50 ml round-bottom flask. The mixture was stirred at 37°C under O_2_ for 24 h before the volatiles were evaporated to dryness on a rotary evaporator. The solid was purified on silica gel chromatography eluted with EtOAc/methanol (10:1 v/v, Rf = 0.35 by TLC). After workup, the desired product was obtained with yield of 20.2 mg (47.1%). ESI-MS: 353.07 (M-) (Fig. 1).

### Cell Culture

Murine RAW264.7 macrophages obtained from the American Type Culture Collection (ATCC) were cultured in α-MEM containing 10% FBS (heat-inactivated) supplemented with 1% penicillin/streptomycin (Hyclone Laboratories, USA). Cells were seeded to culture plates at a density of 10^6^ cells/ml and grown in a humidified chamber with 95% air and 5% CO_2_ at 37°C for at least 12 h to make them attach to the dishes. After that, the cells were stimulated with LPS (200 ng/ml) in the absence or presence of various compounds (dissolved in 0.2%DMSO).

### Cell Viability Assay

Reagent WST-1 was applied to test cell proliferation in accordance with the manufacturer’s instructions. Briefly, the cells grown in 96-well plates were exposed to various concentrations of indicated drugs for 24 h. Afterward, the supernatant was replaced by fresh culture medium containing 10% WST-1. After incubating for an additional 1h, the optical density was determined using a Varioskan Flash microplate reader (USA) at 440 nm.

### NO Detection

Cells grown in triplicate in 96-well plates were stimulated with or without LPS in the absence or presence of related compounds for 24 h, after which the production of NO was measured using the Griess reagent [sulfanilamide solution, N-1-napthylethylenediamine dihydrochloride (NED) solution, standard nitrite sample]. In brief, the cell culture supernatant (50 μl) was transferred to another 96-well plate, followed by sequential allocation of 50 μl sulfanilamide solution and equal volumes of NED solution. The combination was left at room temperature for 5-10 min and then the optical density was read at 540 nm with a Varioskan Flash microplate reader. The concentrations of nitrite were identified from a standard reference curve generated with serial dilutions of standard nitrite sample in culture medium.

### Real-Time RT-PCR Analysis

The mRNA levels of inducible nitric oxide synthase (iNOS), cyclooxygenase-2 (COX-2), IL-1β, IL-6, IL-8, and interferon-β (IFN-β) were detected by real-time reverse transcriptase polymerase chain reaction (RT-PCR). After stimulating RAW cells in the 12-well tissue culture plates for 6 h, TRIzol reagent (Invitrogen, USA) was used to isolate total RNA from cells and 1 μg RNA was converted to cDNA using an iScriptTM cDNA Synthesis Kit (Bio-Rad, USA) according to the protocols provided by the manufacturer. The cDNA was amplified in triplicate using the GoTaq qPCR Master Mix (Promega) on the ViiA 7 real-time PCR system (Life Technologies Corporation, USA). All the primers were obtained from Integrated DNA Technologies (USA) and the sequences were shown in Table 1. The thermal and timing conditions were 95°C (5 min), and 40 cycles of 95°C (15 s) and 60°C (1 min). The Ct values achieved by RT-PCR were normalized according to the level of GAPDH (internal control) and 2-ΔΔCt formula was used to determine their relative quantities.

### Western Blot Analysis

Cells cultured in 6-well tissue culture plates were stimulated for 30 min to detect the protein expression level of mitogen-activated protein kinases (MAPKs) and NF-κB P65, while the iNOS, COX-2, and IL-1β protein levels were analyzed after 24 h treatment. Cells were then washed 3 times with chilled PBS after treatment and lysed with RIPA buffer supplemented with phosphatase and protease inhibitors (Thermo Fisher Scientific). The protein concentration was measured with the BCA Colorimetric Protein Assay Kit (USA). Equal concentrations of cellular protein were separated by 10 or 12% SDS-PAGE and then electro-blotted onto a PVDF membrane. After being blocked with 5% skim milk at room temperature for 1 h, the membranes were incubated with primary antibody overnight at 4°C. Then membranes were washed for three times with TBST (Tris-buffered saline, 0.1%Tween 20) buffer, followed by incubation with appropriate horseradish peroxidase-conjugated secondary antibody for 1 h at room temperature, and washed for 3 times in TBST again. The immunoblots were visualized with the WesternBright ECL Detector (Advansta, USA). The density of the protein bands was quantified by Image-Pro Plus and normalized with GAPDH, β-actin or non-phosphorylated form of protein levels.

### Reactive Oxygen Species (ROS) Assay

The DCFH-DA method was used to measure intracellular ROS generation. After treatment with LPS for 24 h, cells in the 96-well plate were stained with 10 μM DCFH-DA for 30 min at 37°C. Dye fluorescence intensity was monitored at excitation (485 nm) and emission (535 nm). Fluorescent images were photographed using a Leica fluorescence microscope (Leica Microsystems, Germany) and fluorescence intensity was quantified by Image-Pro Plus.

### Fluorescence Microscopy for p65 Nuclear Translocation

Cells were seeded on glass coverslips in a 24-well culture plate and stimulated for 24 h. p65 nuclear translocation was immunofluorescence-labeled using a cellular nuclear factor kappa B (NF-κB) p65 translocation kit (Beyotime Biotech, China) in accordance with the manufacturer’s instructions. Briefly, after fixing with stationary liquid for 10 min, the cells were blocked for 1 h at room temperature and then incubated with the antibody against p65 overnight at 4°C. The next day, the cells were washed with PBS and incubated with the rabbit IgG antibody conjugated with Cy3 for 1 h and stained with 2-(4-amidinophenyl)-6-indolecarbamidine dihydrochloride (DAPI) solution for 5 min. Eventually, images were visualized via a laser confocal scanning microscope (Leica Microsystems) in the dark room at different excitation wavelengths (350 nm for DAPI and 540 nm for Cy3). The two images were combined together to confirm the areas of colocalization.

### Statistical Analysis

The data were presented as mean ± SD of at least three independent experiments and were analyzed via one-way analysis of variance (ANOVA) using Graphpad Prism 5 software. Differences were defined as statistically significant only for *p* < 0.05.

## Results

### Effect of LUA on Cell Viability in RAW Cells

**Fig. 2:**
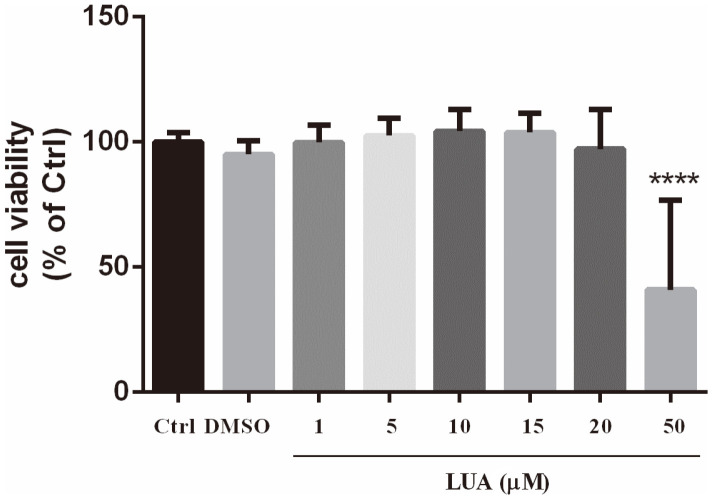
Effect of LUA on cell viability in RAW267.4 cells. The cells were treated with 0.2% DMSO or various concentrations (1-50 μM) of LUA for 24 h. Cell viability was assessed using the WST-1 assay. Each value was presented as mean ± SD (*n* = 10). ****p < 0.0001 vs. 0.2% DMSO-treated cells.

To observe the cytotoxic effect of LUA, RAW264.7 cells were exposed to different concentrations of LUA for 24 h. As shown in Fig. 2, LUA alone at 1-20 μM showed no obvious changes in the cell viability assessed with the WST-1 assay.

### LUA Reduces LPS-Stimulated NO Production and iNOS Expression

**Fig. 3:**
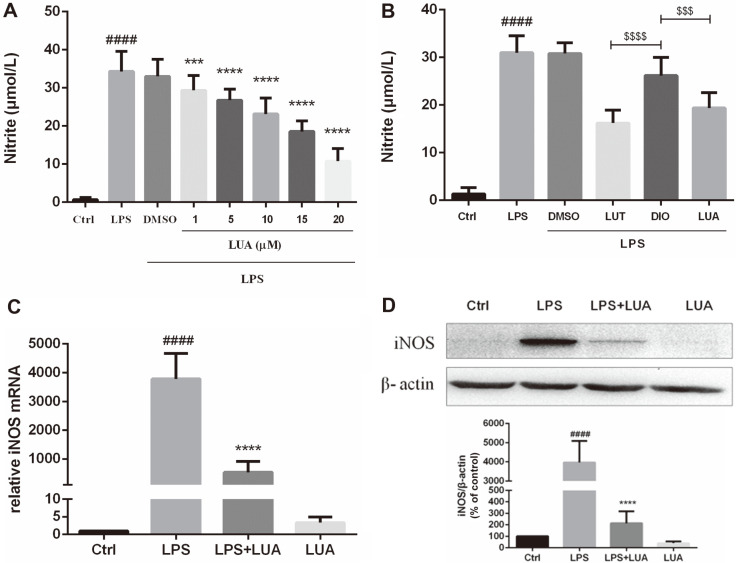
LUA inhibited LPS-induced production of NO in Raw264.7 cells via the downregulation of iNOS expression. (**A**) Cells were incubated with various doses (1-20 μmol/l) of LUA in the absence or presence of LPS. The media were collected after 24 h and assayed for NO production (*n* = 13). (**B**) Cells were incubated with luteolin, diosmetin and LUA at the concentration of 15 uM in the absence or presence of LPS. The media were collected after 24 h and assayed for NO (*n* = 4). (**C** and **D**) Cells were treated with LUA at the concentration of 15 uM in the absence or presence of LPS. (C) After incubation for 6h, total RNA was isolated and RT-PCR was conducted for iNOS mRNA level (*n* = 3). (**D**) After incubation for 24 h, cell lysates were subjected to Western blot analysis with an iNOS or β-actin-specific antibody (*n* = 5). The results were displayed as means ± SD. Data were presented as percentage of control group. ####*p* < 0.0001 vs. Ctrl group; ***p < 0.001, ****p < 0.0001 vs. LPS group. Ctrl: control; LUT: luteolin; DIO: diosmetin; LUA: LUAAPH-1.

To test the effects of LUA on the LPS-induced inflammation, we first determined the concentration-dependent effect of LUA on NO levels in RAW264.7 cells in the presence or absence of LPS for 24 h. As shown in Fig. 3A, LPS stimulation led to marked increases of NO levels, which was effectively suppressed by LUA (0, 1, 5, 10, 15, 20 μM) in a dose-dependent manner. LUA at 15 μM significantly decreased NO by 50%. The concentration of LUA at 15 μM was therefore chosen in the subsequent experiments. We also compared the effects of luteolin, diosmetin and LUA on the production of NO. As shown in Fig. 3B, diosmetin had much weaker effect on suppression of NO production than luteolin and LUA. In accordance with NO production, treatment with LUA (15 μM) also downregulated the LPS-induced upregulation of both mRNA (Fig. 3C) and protein (Fig. 3D) expression of iNOS.

### LUA Inhibits LPS-Induced COX-2 mRNA and Protein Expression

**Fig. 4:**
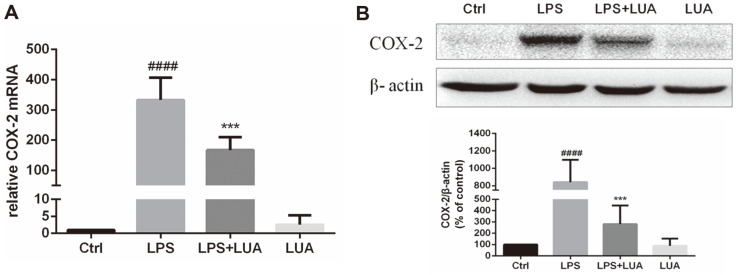
Effects of LUA on LPS-induced COX-2 mRNA (**A**) and protein expression (**B**) in RAW264.7 cells. Cells were stimulated with or without LPS in the presence or absence of LUA (15 μM). (**A**) The COX-2 mRNA expression level was detected by RT-PCR after treatment for 6h (*n* = 4). (**B**) The COX-2 protein expression level was measured by Western blot after stimulation for 24 h (*n* = 4). Data were presented as percentage of control group. The results were displayed as means ± SD. ####*p* < 0.0001 vs. Ctrl group; ***p < 0.001 vs. LPS group. Ctrl: control; LUA: LUAAPH-1.

To further confirm the anti-inflammatory effect of LUA, we determined the effect of LUA on the mRNA and protein expression of COX-2 in RAW264.7 cells stimulated with LPS for 6 h or 24 h. As shown in Figs. 4A and 4B, stimulation with LPS remarkably increased COX-2 protein expression in RAW cells. However, co-treatment with LUA significantly attenuated LPS-induced COX-2 mRNA and protein expressions in the cells.

### LUA Regulates LPS-Induced Expression of Pro-Inflammatory Cytokines

**Fig. 5:**
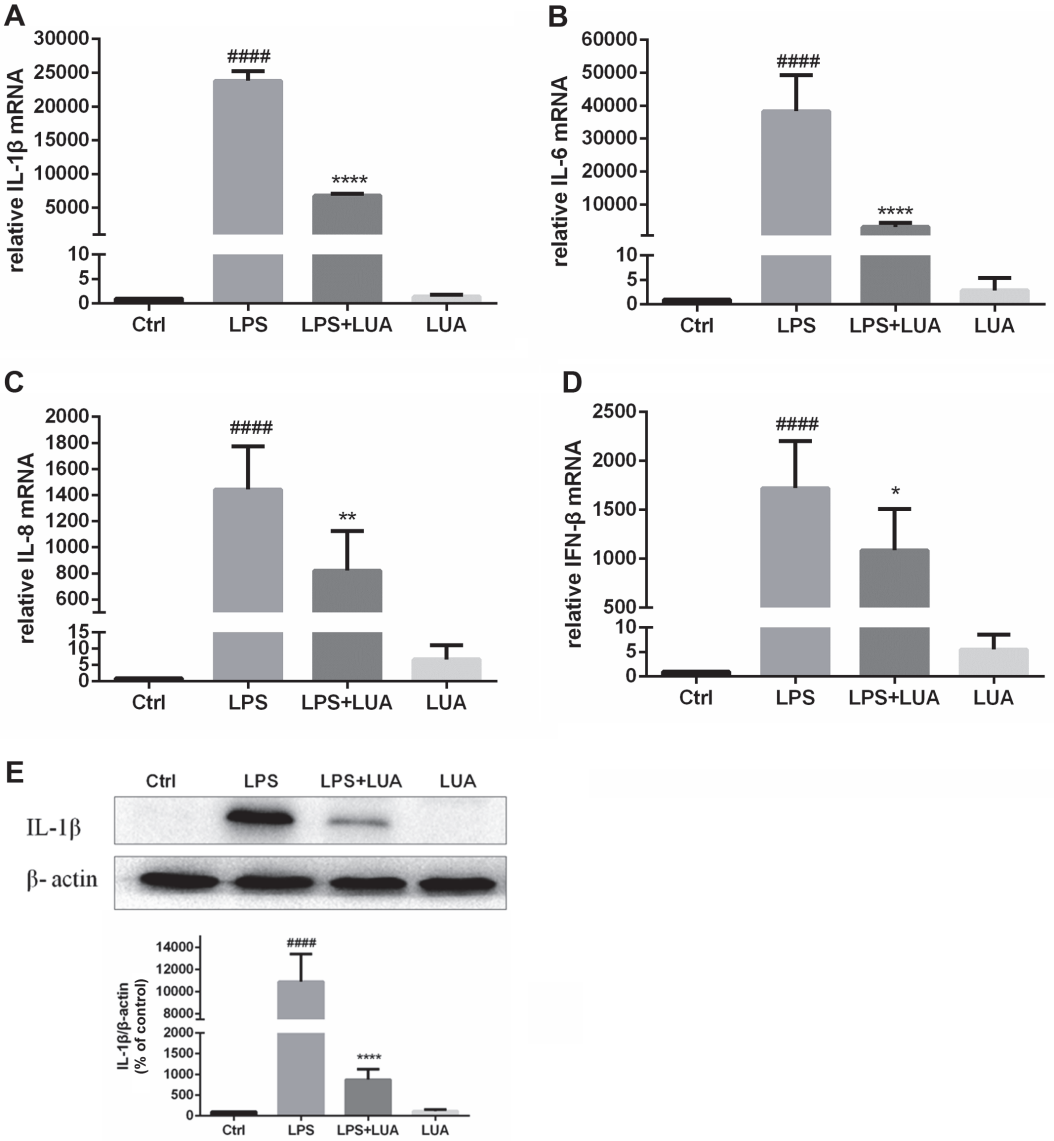
Effects of LUA on LPS-induced pro-inflammatory cytokines in RAW264.7 cells. Cells were treated with LPS in the absence or presence of LUA (15 μM). (**A-D**) Following stimulation with LPS for 6 h, the IL-1β (**A**) (*n* = 3), IL-6 (**B**) (*n* = 6), IL-8 (**C**) (*n* = 5), IFN-β (**D**) (*n* = 4) mRNA expression levels were determined by RT-PCR. (**E**) Following incubation for 24 h, the protein quantity of IL-1β was measured by Western blot (*n* = 4). Data were presented as percentage of control group. The results were displayed as means ± SD. ####*p* < 0.0001 vs. Ctrl group; *p < 0.05, **p < 0.01, ****p < 0.0001 vs. LPS group. Ctrl: control; LUA: LUAAPH-1.

To evaluate the effect of LUA on pro-inflammatory cytokines, the mRNA levels of IL-1β, IL-6, IL-8, and IFN-β were examined by RT-PCR after treatment with LPS and LUA. As shown in Figs. 5A-5D, the mRNA expression of IL-1β, IL-6, IL-8 and IFN-β were significantly elevated at 6 h in response to LPS treatment. As expected, LUA (15 μM) attenuated LPS-induced upregulation of indicated mRNA levels. Moreover, the protein expression of IL-1β was also analyzed at 24 h after LPS and LUA co-incubation. Consistent with RT-PCR results, treatment with LUA (15 μM) suppressed LPS-induced increase in IL-1β protein expression, revealing that the reduction in IL-1β protein expression was associated with a decrease in the corresponding mRNA level (Fig. 5E).

### LUA Suppresses LPS-Evoked Cellular ROS Generation

**Fig. 6:**
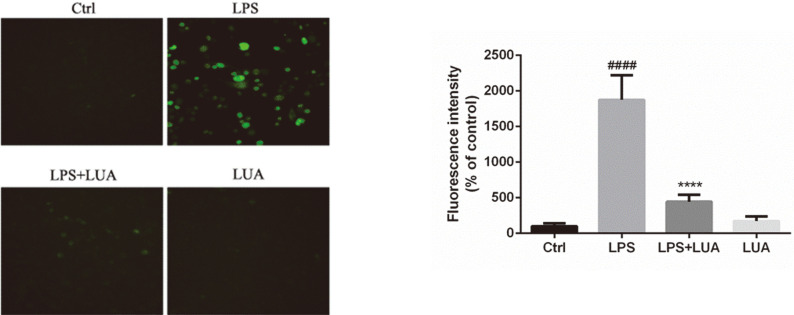
Intracellular ROS levels in RAW264.7 cells. After incubating with LPS in the presence or absence of LUA (15 μM) for 24 h, the cells were exposed to DCFH-DA for 30 min at 37°C. The fluorescence was measured at 485 nm (excitation) and 535 nm (emission). Data were presented as percentage of control group (*n* = 6). The results were displayed as means ± SD. ####*p* < 0.0001 vs. Ctrl group; ****p < 0.0001 vs. LPS group. Ctrl: control; LUA: LUAAPH-1.

ROS production is considered to be involved in the inflammation responses triggered by LPS in macrophages [35, 36]. Therefore, the effect of LUA on intracellular ROS formation was monitored. As shown in Fig. 6, LPS treatment for 24 h induced massive production of intracellular ROS, which was dramatically reduced by the co-treatment with LUA (15 μM).

### LUA Abolishes LPS-Triggered Phosphorylation of MAPKs

**Fig. 7:**
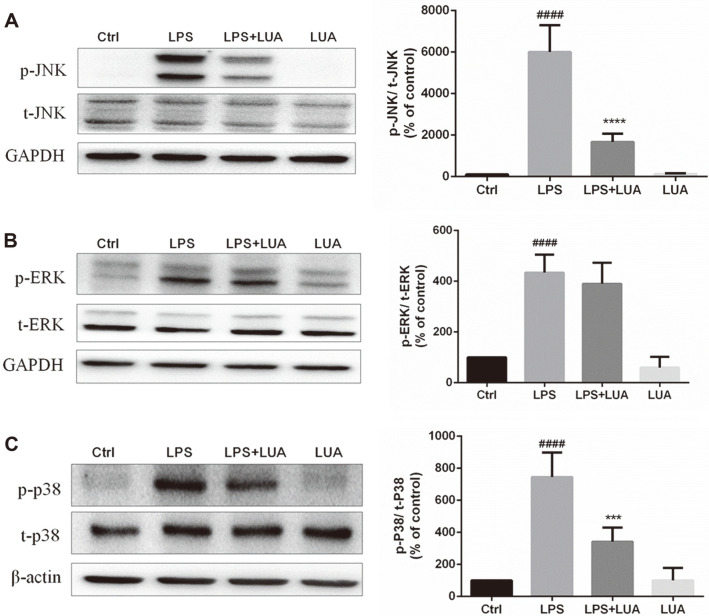
Effects of LUA on LPS-induced MAPK signaling pathways in RAW264.7 cells. Cells were incubated with LPS for 30 min in the absence or presence of LUA (15 μM). Cells lysates were subjected to Western blot for analysis of p-JNK (**A**) (*n* = 4), p-ERK (**B**) (*n* = 5)and p-P38 (**C**) (*n* = 4). The relative abundance of the phosphorylated form to its total protein was quantified. Data were presented as percentage of control group. The results were displayed as means ± SD. ####*p* < 0.0001 vs. Ctrl group; ***p < 0.001, ****p < 0.0001 vs. LPS group. Ctrl: control; LUA: LUAAPH-1.

MAPK signaling pathways play significant roles in the regulation of inflammatory responses [37-41]. Therefore, to investigate the effects of LUA on LPS-induced MAPK signaling, the levels of p-JNK, p-ERK and p-p38 were measured in LPS-stimulated RAW264.7 cells. As shown in Figs. 7A and 7C, without altering total expression levels of MAPKs, treatment with LPS for 30 min resulted in the phosphorylation of JNK and p38, which were abrogated by LUA (15 μM). However, the phosphorylation of ERK was not affected by LUA (Fig. 7B), indicating that LUA strongly blocked the JNK and p38 pathway but not ERK pathway in LPS-stimulated RAW cells.

### LUA Ameliorates LPS-Triggered p65 Phosphorylation and Nuclear Translocation

**Fig. 8:**
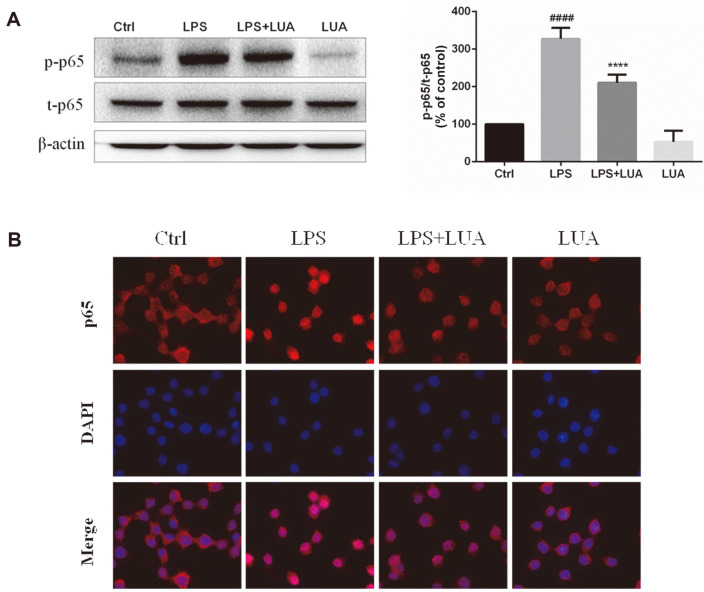
Effects of LUA on LPS-induced NF-κB signaling pathway in RAW264.7 cells. (**A**) Cells were incubated with LPS for 30 min in the absence or presence of LUA (15 μM). Cells lysates were subjected to Western blot for analysis of p-p65 (*n* = 4). The relative abundance of p-p65 to t-p65 was quantified. (**B**) Cells were incubated with LPS for 1 h in the absence or presence of LUA (15 μM). Nuclear translocation of p65 was visualized by immunofluorescence analysis with a confocal laser scanning microscope. Data were presented as percentage of control group. The results were displayed as means ± SD. ####*p* < 0.0001 vs. Ctrl group; ****p < 0.0001 vs. LPS group. Ctrl: control; LUA: LUAAPH-1.

NF-κB is also critically involved in inflammatory processes. The effects of LUA on LPS-stimulated NF-κB activation were shown in Fig. 8. As expected, the phosphorylated NF-κB p65 in RAW cells was markedly upregulated by LPS stimulation for 30 min. In contrast, LUA (15 μM) inhibited the expression of phosphorylated p65 challenged by LPS, with no significant difference of total p65 expression (Fig. 8A). In parallel, p65 nuclear translocation was measured by immunofluorescence using a confocal laser microscope. In line with previous results, the level of nuclear p65 was dramatically increased after LPS stimulation for 1 h, which in turn was markedly suppressed by LUA (15 μM) co-treatment (Fig. 8B).

## Discussion

Flavones, as a subclass of flavonoids, have a basic structure comprising two aromatic rings (A and B) connected through a heterocyclic C-ring. They differ from other flavonoids via the formation of a double bond between C2 and C3, following a carbonyl group at the C4 position on the C-ring. In addition, the absence of a hydroxyl group at the C3 position of C-ring distinguishes flavones from flavonols, another subgroup of flavonoids [42]. Luteolin is a flavone compound characterized by a hydroxyl group attached at the C5, C7, C3′, and C4′ positions of the flavone skeleton. Therefore, B-ring of luteolin, with a catechol structure, can be methylated by COMT. Since COMT can only catalyze the substitution of one hydroxyl in catecholic substrates by one O-methyl group from S-adenosyl-L-methionine, two methylated metabolites of luteolin can be formed, which are called chrysoeriol (3'-O-methylated luteolin) and diosmetin (4'-O-methylated luteolin). However, some studies have revealed that COMT preferred the methylation for 4'-hydroxyl position to 3'-hydroxyl position of luteolin in human primary hepatocyte, HepG2 cells, human liver S9, and rat tissues [28, 29, 31]. The mechanism for regioselectivity of luteolin might be attributed to the more stable binding mode for 4'-O-methylation of luteolin than that for 3'-O-methylation, which was based on combined theoretical investigations including molecular dynamics simulations, ab initio calculations, and quantum mechanics/molecular mechanics computations [43]. It has been reported that COMT-mediated methylation is responsible for the inactivation of endogenous catecholamine neurotransmitters [23, 44, 45]. Current studies have also observed decreased anti-inflammation ability of methylated luteolin metabolites [31], implying the necessity to improve the chemical structure of luteolin for higher bioactivity considering the methylated metabolism in vivo.

AAPH is a water-soluble azo compound that thermally and spontaneously decomposes to yield nitrogen gas (N2) and two carbon-centered radicals (R·). The carbon radicals could either recombine to generate stable products (RR) or react rapidly and directly with molecule oxygen to yield peroxyl radicals (ROO•) [46], which are implicated in the damage of lipids, proteins, DNA and other cellular constituents [47]. Luteolin is also involved in free radical scavenging and has been reported to be capable of eliminating free radicals generated by AAPH [48, 49], which is a peroxy radical generator for antioxidant capacity assays such as the oxygen radical absorbance capacity assay (ORAC) [50]. We measured the ORAC values of luteolin and found that it had potent activity with ORAC value of 6.8 [51]. We further characterized the reaction products of luteolin and AAPH and isolated LUA as the major product. This new compound attracted our attention to a great deal because without affecting the skeleton of flavone, the hydroxyl group at the 3' position was substituted by an ester. Therefore, this flavone will not be methylated by COMT at 3' and is likely to exhibit its function in the original form in the body.

Flavones have a long tradition of being employed as an anti-inflammatory remedy [9, 52, 53]. It has been suggested that 4’-hydroxyl substituent on the B-ring is tightly related to the anti-inflammatory effect of flavonoids [54]. The LPS-activated RAW264.7 macrophage inflammatory model is widely used in the research of inflammation because macrophage is the main cell that regulates the inflammatory response with LPS as an important trigger. A variety of cytokines participate in the inflammatory response after the stimulation of LPS in RAW264.7 cells. We further investigated the effects of LUA on inflammation in RAW264.7 cells. As expected, with no cytotoxicity up to the concentration of 20 μM, LUA dose-dependently inhibited the production of NO in macrophages stimulated with LPS. NO production was also compared after LPS treatment in the presence of luteolin, diosmetin and LUA at the concentration of 15 μM. It was found that although treatment with luteolin inhibited LPS-induced NO production to a large extent, its methylated derivative diosmetin was not as effective as luteolin, indicating the much weaker anti-inflammatory effect of luteolin in the metabolic environment of the organism. There results were in agreement with previous research [31]. However, compared with diosmetin, LUA was superior in the suppression of NO, suggesting the possible better anti-inflammatory effect of LUA than luteolin in vivo. The reduction of NO production was likely attributed to the transcriptional level as indicated by the attenuated expression of iNOS mRNA and protein expression. In addition, LUA also substantially down regulated the production of pro-inflammatory molecules including COX-2, IL-1β, IL-6, IL-8, and IFN-β, which was similar to the anti-inflammatory effects of luteolin [55-58].

Notably, accumulated evidence indicates that inflammation and oxidative stress are highly related and orchestrated between each other, creating a vicious cycle in various pathophysiological conditions [59-61]. On one hand, inflammatory cells can liberate more ROS at the inflammatory lesion, leading to exaggerated oxidative stress [59, 62]. On the other hand, a number of ROS can provoke an intracellular signaling cascade that augments pro-inflammatory gene expression. Earlier studies have demonstrated that luteolin suppresses the ROS generation [15], thus regulating the homeostasis between oxidants and antioxidants [18, 20, 63-65]. In our study, LPS-induced inflammation was accompanied by large amounts of ROS generation in the cells as reported [66, 67], whereas ROS were prominently repressed by LUA, suggesting that LUA also exerted its anti-inflammatory effect by removing intracellular ROS.

It is well known that MAPK signaling pathways play crucial roles in regulating the inflammatory signaling from cell surface to nucleus and coordinating induction of different genes encoding for inflammatory mediators [41, 68-70]. It remains controversial whether luteolin is responsible for the inhibition of MAPK signaling pathway in RAW cells stimulated by LPS. Chen *et al*. discovered that treatment of RAW cells with luteolin only decreased JNK phosphorylation and its downstream signaling [71]. Intriguingly, instead of inhibiting MAPK pathway, Lee *et al*. found that luteolin upregulated the phosphorylation of these enzymes in LPS-induced RAW cells [55]. Our results showed that exposure of RAW cells to LPS increased the phosphorylation of p38, ERK and JNK respectively. We further demonstrated that LUA markedly suppressed the phosphorylation of JNK and p38 stimulated by LPS without changing the total protein levels of MAPKs, indicating that LUA specifically inhibited the phosphorylation of JNK and p38 but not the synthesis of these two MAPKs. In contrast, LUA did not affect the phosphorylation of ERK elicited by LPS. Thus, we presumed that LUA-mediated suppression of LPS-induced inflammation in RAW cells was JNK and p38 dependent.

As an important nuclear transcription factor, NF-κB is also a key mediator of inflammation. In the inactive mode, the NF-κB dimer (p50/p65) exists in the cytosol and is bound to IκBα, an inhibitory protein. The presence of several stimuli, including LPS, leads to the phosphorylation and degradation of IκBα, followed by p65 phosphorylation and translocation of NF-κB into the nucleus, where NF-κB subsequently binds to corresponding sites and activates the transcription of many pro-inflammatory genes ultimately [72-75]. It has been noted that LPS-induced ROS generation can also transduce signals to activate NF-κB signaling cascades in LPS-stimulated macrophages [76]. Several previous studies have provided evidence that luteolin mediated the anti-inflammatory effect by blocking the NF-κB signaling pathway [55, 57, 77]. In line with these findings, in the present study, LUA significantly abolished LPS-induced p65 phosphorylation and the subsequent accumulation of p65 in the nucleus without affecting the expression of total p65. Therefore, it is expected that the anti-inflammatory property of LUA also attributed to the inhibition of NF-κB signaling pathway.

**Fig. 9:**
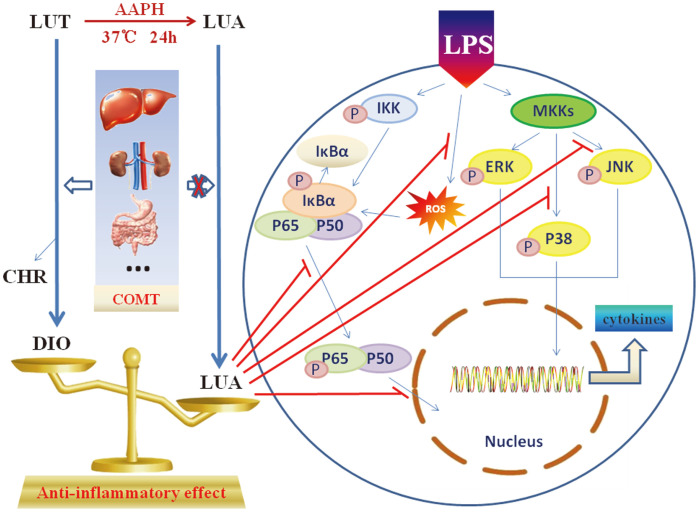
A schematic model that proposes the synthesis procedure, advantages as well as the potential contribution of LUA in anti-inflammatory signaling pathways in LPS-activated RAW264.7 macrophages. LUT: luteolin; LUA: LUAAPH-1; AAPH: 2,2'-azobis(2-amidinopropane) dihydrochloride; DIO: diosmetin; CHR: chrysoeriol; COMT: catechol-O-methyltransferases; LPS: lipopolysaccharide; IKK: inhibitor-κB kinase; IκBα: inhibitor kappa B alpha; MKKs: MAPK kinases; ROS: reactive oxygen species.

In conclusion, we described a novel compound (LUAAPH-1) generated by the chemical reaction between luteolin and AAPH, which was characterized by several unique features. First of all, compared to luteolin, LUA could escape COMT-catalyzed methylation, thus affording the potential to exert its functions in the original form when administrated in the organism. Secondly, the new compound displayed highly superior anti-inflammatory activities than the methylated derivative of luteolin in macrophages and the underlying mechanisms might be the inhibition of LPS-activated JNK, P38 and NF-κB signaling pathways (Fig. 9). The findings suggested the new compound to be a potentially promising therapy in inflammation disorders and related diseases. However, more investigation into the therapeutic effects of the new compound in vivo are still required for the potential clinical applications.

## Figures and Tables

**Table 1:** Primers for real-time RT-PCR [78].

Name	Sequences(5’→3’)
GAPDH	Forward: ACCCCAGCAAGGACACTGAGCAAG
	Reverse: GGCCCCTCCTGTTATTATGGGGGT
iNOS	Forward: GCTCGCTTTGCCACGGACGA
	Reverse: AAGGCAGCGGGCACATGCAA
COX-2	Forward: GGGCTCAGCCAGGCAGCAAAT
	Reverse: GCACTGTGTTTGGGGTGGGCT
IL-1β	Forward: GCCTCGTGCTGTCGGACCCATAT
	Reverse: TCCTTTGAGGCCCAAGGCCACA
IL-6	Forward: AGACAAAGCCAGAGTCCTTCAGAGA
	Reverse: GCCACTCCTTCTGTGACTCCAGC
IL-8	Forward: TTGCCTTGACCCTGAAGCCCCC
	Reverse: GGCACATCAGGTACGATCCAGGC
IFN-β	Forward: GGATCCTCCACGCTGCGTTCC
	Reverse: CCGCCCTGTAGGTGAGGTTGA
